# Identification of the minimal region in lipase ABC transporter recognition domain of *Pseudomonas fluorescens *for secretion and fluorescence of green fluorescent protein

**DOI:** 10.1186/1475-2859-11-60

**Published:** 2012-05-11

**Authors:** Yeonwoo Park, Yuseok Moon, Jungmin Ryoo, Nayeon Kim, Hyounghoon Cho, Jung Hoon Ahn

**Affiliations:** 1 Korea Science Academy of KAIST, 899 Tanggam 3-Dong, Busanjin-Gu, Busan, 614-822, Korea; 2 Department of Microbiology and Immunology, Medical Research Institute, Pusan National University School of Medicine, Yangsan, 626-813, Korea

**Keywords:** Green fluorescent protein (GFP), Lipase ABC transporter recognition domain (LARD), ABC transporter, *Pseudomonas fluorescens*, Secretion/chaperon domain, β-roll

## Abstract

**Background:**

TliA is a thermostable lipase secreted by the type 1 secretion system (T1SS) of *Pseudomonas fluorescens*. The secretion is promoted by its secretion/chaperone domain located near the C-terminus, which is composed mainly of four Repeat-in-Toxin (RTX) repeats. In order to identify the minimal region of TliA responsible for its secretion, five different copies of the secretion/chaperone domain, each involving truncated N-terminal residues and a common C-terminus, were acquired and named as lipase ABC transporter recognition domains (LARDs). Each LARD was fused to epidermal growth factor (EGF) or green fluorescent protein (GFP), and the secretion of EGF-LARD or GFP-LARD fusion proteins was assessed in *Escherichia coli* with ABC transporter.

**Results:**

Among the fusion proteins, GFP or EGF with 105-residue LARD3 was most efficiently secreted. In addition, GFP-LARD3 emitted wild type GFP fluorescence. Structurally, LARD3 had the 4 RTX repeats exposed at the N-terminus, while other LARDs had additional residues prior to them or missed some of the RTX repeats. LARD3 was both necessary and sufficient for efficient secretion and maintenance of GFP fluorescence in *E. coli*, which was also confirmed in *P. fluorescens* and *P. fluorescens ▵tliA*, a knock-out mutant of *tliA*.

**Conclusion:**

LARD3 was a potent secretion signal in T1SS for its fusion flanking RTX motif, which enhanced secretion and preserved the fluorescence of GFP. LARD3-mediated secretion in *E. coli* or *P. fluorescens* will enable the development of enhanced protein manufacturing factory and recombinant microbe secreting protein of interest *in situ*.

## Background

The type 1 secretion system (T1SS) secretes polypeptides to the extracellular medium in gram-negative bacteria [1]. T1SS is composed mainly of ATP-binding cassette (ABC) transporter, which is comprised of ABC protein [2], adaptor or membrane fusion protein (MFP) [3], and outer membrane protein (OMP) [4]. The ABC protein captures the substrate polypeptide and transports it through the contiguous channel formed by MFP and OMP by coupling ATP hydrolysis [5-7]. The MFP-OMP channel penetrates both gram-negative membranes simultaneously, leaving no periplasmic intermediates [8]. T1SS is also referred to as a signal peptide-independent secretion system because the secretion is mediated by the C-terminal region of a substrate polypeptide instead of the signal sequence [9].

TliA is a 476 aa thermostable lipase encoded in the lipase operon of *Pseudomonas fluorescens*. TliA is secreted by a dedicated T1SS composed of TliDEF, an ABC transporter encoded in the upstream of TliA within the lipase operon [10]. TliA is exported by TliDEF in high efficiency at 25°C, which is the optimum growth temperature of *P. fluorescens*[10]. By homology, TliA is also exported by PrtDEF, the ABC transporter of *Erwinia chrysanthemi,* at 37°C [11]. TliA has a lipase activity domain, a hinge region, a secretion/chaperone domain, and a calcium-binding domain (4 RTX repeats) in residues 1–268, 269–278, 279–476, and 373–417, respectively [11].

Several T1SS substrate polypeptides have been widely studied. Those include HlyA from *Escherichia coli*[12,13], PrtB and PrtC from *E. chrysanthemi*[14,15], AprA_PA_ from *Pseudomonas aeruginosa*[16], and TliA from *P. fluorescens*[10]. These substrate polypeptides share common structures such as an extreme C-terminal motif [17], a hydrophobic five-residue sequence motif (VTLIG) [18,19] and a Repeat-in-Toxin (RTX) motif, GGxGxDxUx repeats (x: any amino acids; U: large hydrophobic residues such as L, I, or F). According to the crystal structures of AprA_PA_[20,21] and PrtA_SM_[22,23], the RTX motif forms a β-roll structure in which two RTX repeats constitute a complete turn involving a short parallel β-sheet. The RTX motif has also been referred to as a calcium-binding domain [9,16,24,25], because Ca^2+^ ions are required for the stabilization of the β-roll structure [26]. The exact role of the RTX motif has not been pinpointed, but it is known to affect receptor binding [27], enhance secretion [28], act as an internal chaperone [21], and increase protein levels in the cytoplasm [29]. T1SS substrate polypeptides have no strictly-defined residues required for secretion, but the overall structure including the RTX motif determines the secretion.

In this research, the minimal region in TliA responsible for its secretion was investigated. Five different copies of the secretion/chaperone domain, the C-terminal region of TliA involving the RTX motif, were obtained. These different lengths of polypeptides, defined as the lipase ABC transporter recognition domain (LARD), were attached to the C-terminus of epidermal growth factor (EGF) or green fluorescent protein (GFP). The fusion proteins were expressed with appropriate T1SS in *E. coli*, *P. fluorescens*, and *P. fluorescens ΔtliA*, a knock-out mutant lacking *tliA* in its genome. As a result, LARD3 was necessary and sufficient for efficient secretion. In addition, several observations suggested the RTX motif as both a secretion enhancer and an internal chaperone.

## Results

### Construction of GFP-LARD and EGF-LARD fusion proteins

**Figure 1:**
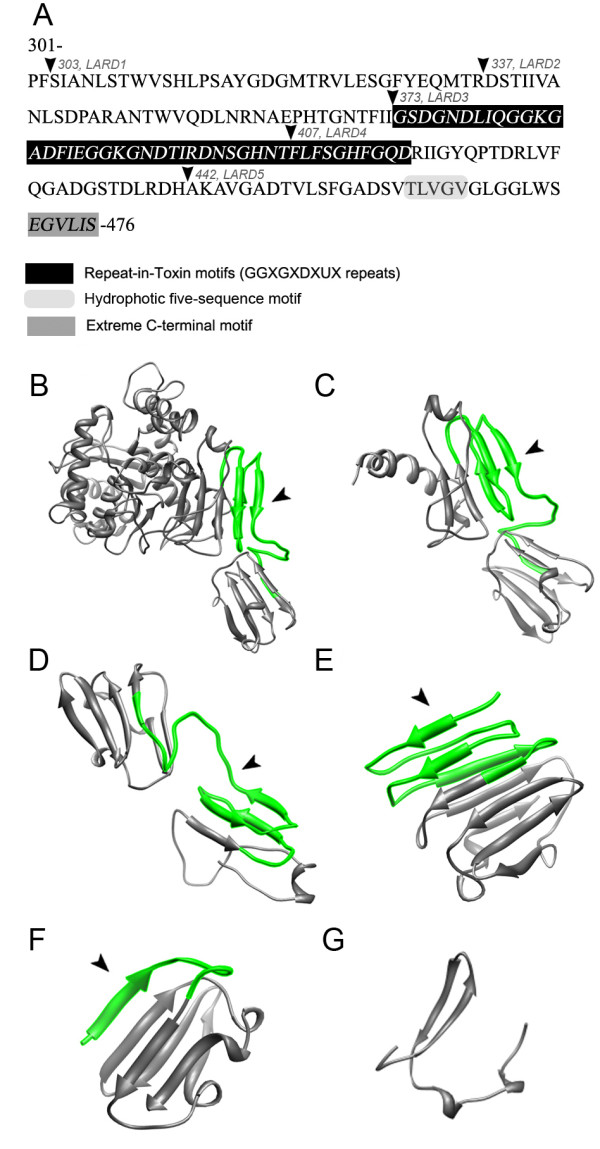
**The features and structures of LARDs. A.** The secretion/chaperone domain of TliA (residues 301–476). The residues and PCR amplification sites of LARDs are also shown overlapped. TliA has a calcium-binding domain (373–416) comprised of four GGxGxDxUx repeats, hydrophobic five-sequence motif and an extreme C-terminal motif, EGVLIS. The three-dimensional structures of each LARDs were predicted by SWISS-MODEL structural modeling according to their residues and displayed by UCSF Chimera (http://www.cgl.ucsf.edu/chimera/) [30]. The RTX motif is indicated by the arrowhead. **B.** TliA, C-G. LARD1 to 5. Note that LARD3 has the RTX motif exposed at the N-terminus. The protein data bank (PDB) IDs for the templates used to predict each structure are as follows: TliA, 2z8z_A; LARD1, 2z8z_A; LARD2, 2qub_A; LARD3, 2zvd_C; LARD4, 2qub_A; LARD5; 2zvd_C.

LARD1 to 5 were constructed by PCR amplifying the secretion/chaperone domain of TliA from residues 303, 337, 373, 407, and 442 to the C-terminus (residue 476), respectively. The features of the secretion/chaperone domain and LARDs are shown (Figure 1A). LARDs were fused to the C-terminus of GFP to construct GFP-LARD fusion proteins with a Factor Xa protease cleavage site as a linker. EGF-fusion proteins were constructed in a similar manner as GFP-fusion proteins. The three-dimensional structures of LARDs predicted by SWISS-MODEL structural modeling program [30] are also represented (Figure 1B). LARD1 (174 *aa*) included 70 additional residues prior to the 4 RTX repeats. Those residues were partly removed in LARD2 (140 *aa*) and completely removed in LARD3 (104 *aa*). As a result, the RTX motif was exposed at the N-terminus of LARD3. Three out of 4 RTX repeats were removed in LARD4 (70 *aa*), and LARD5 (35 *aa*) was deprived of any common structures except for the extreme C-terminal motif.

### Expression and secretion of EGF-LARDs in *E. coli*

**Figure 2:**
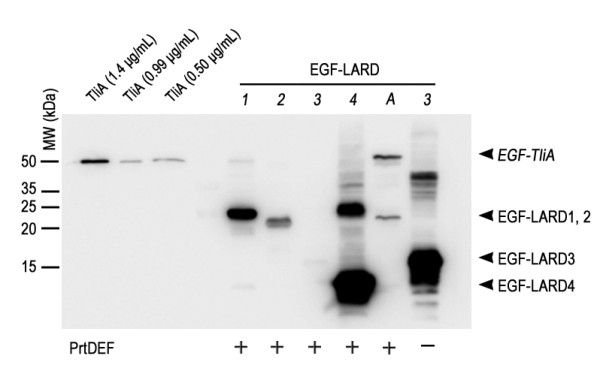
**The intracellular expression of EGF-LARDs in*E. coli*XL1-Blue.** Each *E. coli* XL1-Blue culture harboring one of pEGF-LARD1-4 (lanes *1*–*4*) or pEGF-TliA (lane *A*) with pEcPrtDEF (+) or pACYC-184 (−) was incubated with 0.05 mM IPTG, 50 μg/ml ampicillin, and 34 μg/ml chloramphenicol in 2 ml glass tubes at 37°C. The cells were isolated from the extracellular medium by double centrifugation, underwent SDS-PAGE in 15% gel, and analyzed by western blotting using anti-LARD antibodies. The plasmid pACYC-184 was used as a negative control for pEcPrtDEF which encodes PrtDEF. Purified TliA at a concentration of 99 μg/ml was used as an index after dilution. The arrowheads indicate the size of each EGF-LARD.

The expression and secretion of EGF-LARD1 to 5 and EGF-TliA were examined in *E. coli* XL1-Blue with or without PrtDEF, the ABC transporter of *E. chrysanthemi*. The expression of EGF-fusion proteins, as analyzed by western blotting, was heterogeneous (Figure 2). EGF-LARD3 was undetected in the cell when it was co-expressed with PrtDEF. However, it was detected inside the cell when PrtDEF was absent. The same result was also observed in *E. coli* MM294. Because it is close to the original strain *E. coli* K12 [31], exhibiting wild-type phenotype, and showed strong fluorescence when transformed with GFP gene in our experiment, *E. coli* MM294 was used together with XL1-Blue throughout the experiments. However, we do not draw any conclusion by contrasting the results obtained from the two strains as our purpose in this research is to draw a generalization that applies to a wide range of species.

**Figure 3:**
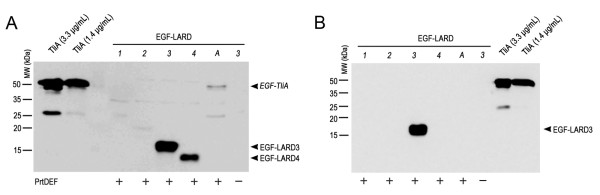
**The extracellular secretion of EGF-LARDs in*E. coli*XL1-Blue and MM294. A.** The secretion of EGF-LARDs in *E. coli* XL1-Blue. Each culture harboring one of pEGF-LARD1-4 (lanes *1*–*4*) or pEGF-TliA (lane *A*) with pEcPrtDEF was centrifuged twice, and the supernatant was collected. The growth conditions were identical to those in Figure 2. SDS-PAGE with 15% gel and western blotting with anti-LARD antibodies were performed on the supernatant. The (+) sign indicates the presence of PrtDEF. The (−) sign indicates the absence of PrtDEF (pACYC-184 was used as a negative control). Purified TliA at 99 μg/ml concentration was used as an index after dilution. **B.** The secretion of EGF-LARDs in *E. coli* MM294. The methods used here were identical to those used in (A).

For extracellular secretion, EGF-LARD3, EGF-LARD4 and EGF-TliA were secreted from *E. coli* XL1-Blue (Figure 3A) while only EGF-LARD3 was secreted from *E. coli* MM294 (Figure 3B). Since EGF-LARD3 was detected only in the extracellular medium when the cells co-expressed PrtDEF, and was detected solely in the cytoplasm when the cells did not express PrtDEF, it was evident that secretion occurred through the ABC transporter instead of other possible pathways such as cell rupture. The concentration of the secreted EGF-LARD3 was about 3.3 μg/ml as determined by comparing to the purified lipase solution.

### Expression and secretion of GFP-LARDs in *E. coli*

**Figure 4:**
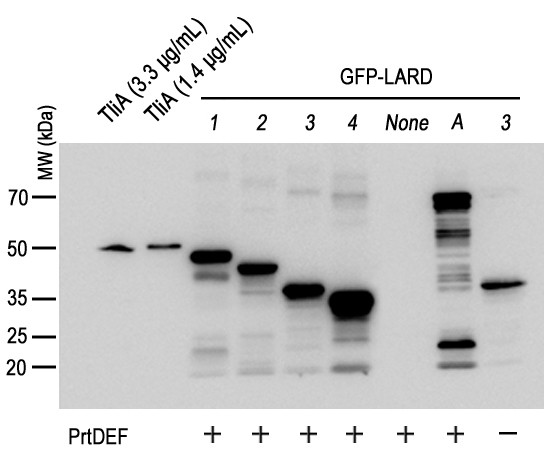
**The intracellular expression of GFP-LARDs in*E. coli*XL1-Blue.** Each *E. coli* XL1-Blue culture harboring one of pGFP-LARD1-4 (lanes *1*–*4*), pGFP-TliA (lane *A*) or pGFP-223 (lane *None*) with pEcPrtDEF (+) or pACYC-184 (−) was grown with 50 μg/ml ampicillin and 34 μg/ml chloramphenicol in a 2 ml glass tube at 37°C (IPTG was not added). The culture was centrifuged twice, and the pellet was isolated. SDS-PAGE with 10% gel and western blotting with anti-LARD antibodies were performed on the pellet. Purified TliA at a concentration of 99 μg/ml was used as an index after dilution.

**Figure 5:**
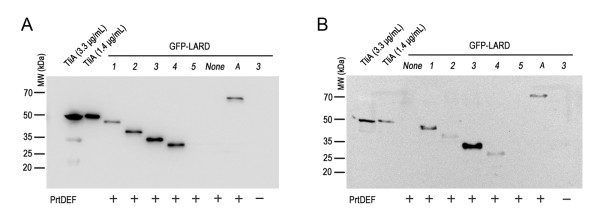
**The extracellular secretion of GFP-LARDs in*E. coli*XL1-Blue and MM294. A.** The secretion of GFP-LARDs in *E. coli* XL1-Blue. Each *E. coli* XL1-Blue culture harboring one of pGFP-LARD1-5 (lanes *1*–*5*), pGFP-TliA (lane *A*) or pGFP-223 (lane *None*) with pEcPrtDEF (+) or pACYC-184 (−) was centrifuged twice, and the supernatant was collected. The growth conditions were identical to those listed in Figure 4. SDS-PAGE with 10% gel and western blotting with anti-LARD antibodies were performed on the supernatant. Purified TliA at a concentration of 99 μg/ml was used as an index after dilution. **B.** The secretion of GFP-LARDs in *E. coli* MM294. The methods used here were identical to those listed in A. The signs are also identical to those of A.

The intracellular expression of GFP-LARDs in *E. coli* was analyzed by western blotting (Figure 4). GFP-LARDs and GFP-TliA were uniformly expressed by *E. coli* cells harboring pGFP-LARD1 to 5 or pGFP-TliA, each with pEcPrtDEF (the vector harboring *prtDEF*). The secretion pattern of different GFP-LARD fusion proteins was analyzed in *E. coli* XL1-Blue (Figure 5A) and *E. coli* MM294 (Figure 5B). In contrast to EGF, GFP was secreted by many types of LARDs except for LARD5. Among the fusion proteins, GFP-LARD3 showed the greatest concentration in the extracellular medium. This pattern was more evident in *E. coli* MM294 than in XL1-Blue, the concentration of GFP-LARD3 in the former being approximately 10 μg/ml.

**Figure 6:**
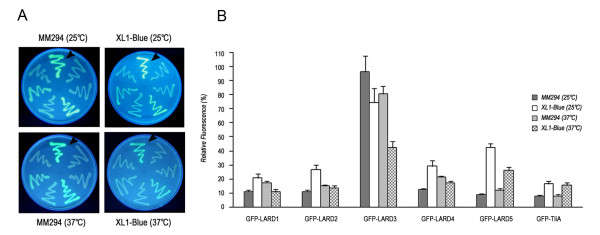
**The GFP fluorescence of recombinant*E. coli*colonies at different temperatures. A.** GFP fluorescence detected on LB plates. Single colonies of *E. coli* XL1-Blue and MM294, each harboring one of the designated plasmids, were streaked on LB plates and grown at 25°C or 37°C. They were subsequently analyzed under UV light. Starting from the culture designated by the arrowhead in a clockwise direction, the plasmids content is as follows: pGFP-223; pGFP-LARD1-5; and pGFP-TliA. **B.** GFP fluorescence assay. The cultures of *E. coli* XL1-Blue and MM294 in A were grown in liquid LB. The cells were washed and analyzed under excitation of 485 nm and emission of 535 nm. The relative fluorescence of each culture compared to that of the culture harboring pGFP-223 was plotted on the graph in a percentage scale (%).

The GFP fluorescence of different GFP-LARD fusion proteins was analyzed under UV light (Figure 6A) and quantitatively measured by fluorescence spectroscopy (Figure 6B). At 25°C which is the optimum growth temperature of *P. fluorescens*, the host species of TliA from which LARDs are derived, GFP-LARD3 showed the highest fluorescence in both *E. coli* XL1-Blue and MM294, exhibiting 96% that of the wild type GFP in MM294. As the temperature increased from 25°C to 37°C, the fluorescence decreased for GFP-LARD3. Other GFP-LARD fusion proteins showed a similar decrease in fluorescence in *E. coli* XL1-Blueand MM294.

Further analyses on the expression and secretion of GFP-LARDs in *P. fluorescens* were undertaken with GFP-LARD3 and GFP-TliA which were suitable for detection in the western blotting and lipase activity plate, respectively. The GFP-TliA was detected easily on the tributyrin plate when it was secreted by ABC transporter, showing halos around colonies.

### Expression and secretion of GFP-LARD3 and GFP-TliA in *P. fluorescens*

**Figure 7:**
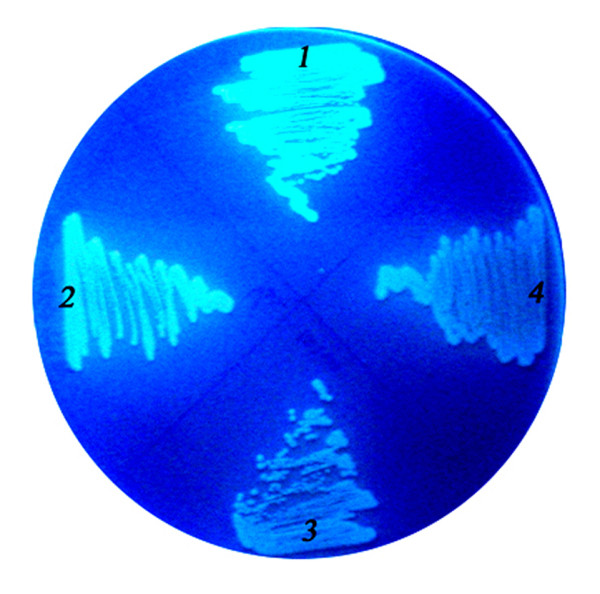
**The GFP fluorescence of recombinant*P. fluorescens*colonies.** The recombinant *P. fluorescens* cells harboring appropriate plasmids were cultured on an LB plate, and the GFP fluorescence was analyzed using UV light. Label 1, pDSK-TliDEF-GFP-LARD3 (pDX-GFP-LARD3); label 2, pDX-GFP-223; label 3, pDX-TliA; label 4, pDX-GFP-TliA.

LARDs were derived from a lipase of *P. fluorescens*. To check whether GFP-LARD fusion proteins were secreted better in the original host, the fusion proteins and an ABC transporter were introduced into *P. fluorescens*. In the former experiments, the GFP-fusion proteins were secreted in *E. coli* through a two-vector system in which the coding sequences of GFP-fusion proteins were inserted in pKK223 and that of the ABC transporter, PrtDEF, was inserted in pACYC184. The plasmids used for *E. coli* could not be used in *P. fluorescens*, so a broad host range vector was constructed; *tliDEF*, the ABC transporter encoded in the lipase operon of *P. fluorescens,* was inserted into pDSK-519 together with the coding sequences of GFP-LARD3 and GFP-TliA. A similar result obtained from *E. coli* (Figure 6) was also observed when *P. fluorescens* cells harboring above plasmids were assayed for GFP fluorescence (Figure 7). As expected, in *P. fluorescens*, GFP-LARD3 had a comparable GFP fluorescence to that of an intact GFP. *P. fluorescens* harboring pDX-GFP-TliA showed a low fluorescence comparable to that of wild type *P. fluorescens*.

**Figure 8:**
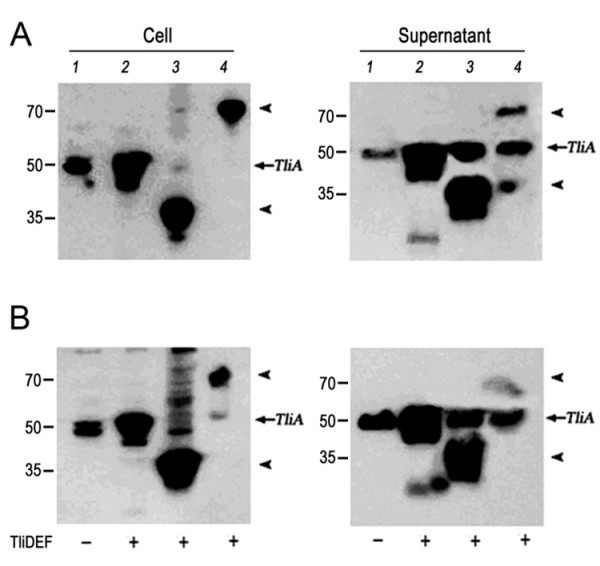
**The expression and secretion of the recombinant proteins in*P. fluorescens*. A.** The intracellular expression and secretion of GFP-TliA and GFP-LARD3 in *P. fluorescens* cultured in LB were detected by western blotting using anti-LARD antibodies. The *P. fluorescens* cells expressing the designated proteins with (+) or without (−) TliDEF were isolated from two different extracellular media by double centrifugation. **B.** The same expression was performed in 2× LB (double concentrated LB). Lane 1, pDSK-TliA; lane 2, pDX-TliA; lane 3, pDX-GFP-LARD3; lane 4, pDX-GFP-TliA. The arrows indicate the sizes of GFP-LARD3 (38.2 kDa), TliA (49.9 kDa), and GFP-TliA (77.4 kDa). Notice the intrinsic TliA indicated by the arrows.

The intracellular and extracellular expressions of GFP-LARD3 and GFP-TliA in *P. fluorescens* were analyzed by western blotting using anti-LARD antibodies (Figure 8). The *P. fluorescens* cells harboring pDSK-TliA, pDX-TliA, pDX-GFP-LARD3, and pDX-GFP-TliA were grown in LB or 2× LB media. The intracellular expression was uniform for each protein in each growth condition. The intrinsic TliA (encoded by the genomic *tliA*) was detected in every extracellular medium, as indicated by the arrow. Here, TliA was also secreted by the intrinsic ABC transporter in the lipase operon of *P. fluorescens* (Figure 8, lane 1). GFP-LARD3 was more efficiently exported than GFP-TliA in *P. fluorescens*, as was the case in *E. coli*. The secretion efficiency was generally higher when the fusion proteins were exported by TliDEF in *P. fluorescens* than by PrtDEF in *E. coli*. The intrinsic TliA was secreted more favorably by TliDEF in that some fractions of GFP-LARD3 and GFP-TliA were left inside the cells while all intrinsic TliA was found outside the cells (Figure 8, lane 3 and 4). Further analyses were undertaken with *P. fluorescens ΔtliA* to eliminate the intrinsic TliA from the extracellular medium.

### Expression and secretion of GFP-LARD3 and GFP-TliA in *P. fluorescens ΔtliA*

**Figure 9:**
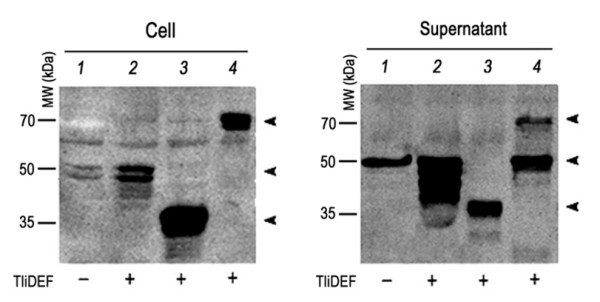
**The expression and secretion of the recombinant proteins in*P. fluorescens ΔtliA*.** The intracellular expression and secretion of GFP-TliA and GFP-LARD3 were analyzed by western blotting using anti-LARD antibodies. The cells were isolated from the growth medium by double centrifugation. Lane 1, pDSK-TliA; lane 2, pDX-TliA; lane 3, pDX-GFP-LARD3; lane 4, pDX-GFP-TliA. The arrows indicate the sizes of GFP-LARD3 (38.2 kDa), TliA (49.9 kDa), and GFP-TliA (77.4 kDa).

*P. fluorescens ΔtliA,* the genomic *tliA*-deficient strain, was used to detect the intracellular expression and extracellular secretion of GFP-LARD3 and GFP-TliA (Figure 9). Cells harboring pDSK-TliA, pDX-TliA, pDX-GFP-LARD3, and pDX-GFP-TliA were analyzed by western blotting. The patterns were identical to those observed in the wild type *P. fluorescens*, except that the intrinsic TliA was absent in the extracellular medium of pDX-GFP-LARD3. GFP-LARD3 was detected inside the cell and in the extracellular medium without TliA. However, although the genomic *tliA* was deleted, both TliA and GFP-TliA were found in the extracellular medium of the cells that expressed only GFP-TliA. When the same intracellular and extracellular media were analyzed using anti-GFP antibodies, GFP was found as its monomeric size in the extracellular medium while only the intact GFP-TliA was detected inside the cell (data not shown). It appears that GFP-TliA was degraded by proteolysis after secretion. Experiments with *P. fluorescens ΔprtA,* which was constructed recently in our laboratory, showed that GFP-TliA was indeed degraded by the protease PrtA which is encoded in the lipase operon and is also secreted by TliDEF [10]. The degradation of extracellular GFP-TliA was not observed in *E. coli* as shown in previous figures.

## Discussion

In this research, LARD has been reported to be a secretion signal capable of exporting GFP or EGF through the gram-negative T1SS. LARD1 to 5 were acquired from the secretion/chaperone domain of TliA, and they involved part of the homologous structures essential for secretion, such as the RTX repeats, hydrophobic five-residue sequence motif and an extreme C-terminal motif. LARDs were attached to GFP or EGF, and the fusion proteins were expressed in *E. coli* and *P. fluorescens* with PrtDEF or TliDEF. The secretion was observed with different LARD fusion proteins, among which EGF-LARD3 and GFP-LARD3were most efficient in the secretion of EGF and GFP. In addition, GFP-LARD3 retained its native GFP fluorescence, while the other fusion proteins had a diminished fluorescence.

The GFP fluorescence was preserved particularly in GFP-LARD3. Note that LARD3 had the 4 RTX repeats exposed at the N-terminus, while others had additional residues prior to them or missed several repeats. It appeared that in GFP-LARD3, the RTX repeats had preserved GFP by structurally separating it from the remaining residues of LARD3. It is yet uncertain whether or not RTX repeats form a stable β-roll structure or remain intrinsically disordered in the cytosol, but in either case, they were able to preserve GFP by acting like a structural barrier. In support of this hypothesis, Blenner *et al.* provided evidence of the RTX motif as a distinct domain that can be folded or unfolded independently to the rest of the polypeptide [32]. Either as a β-roll or as an intrinsically disordered structure, the RTX repeats were able to separate GFP from the secretion signal.

The exact secretion mechanism of T1SS is still being researched. Relevant findings in understanding the mechanism of T1SS include: 1) that the substrate polypeptides sequentially form a multi-protein complex with the ABC protein, MFP, and OMP prior to secretion [33]; and 2) RTX repeats are intrinsically disordered in the calcium-free cytosol and keep the substrate polypeptide translocation-competent [32,34]. Once exported to the calcium-rich extracellular environment, RTX re-peats structuralize under conditions involving entropic stabilization and become an internal chaperone. This spontaneous calcium-induced folding in the extracellular medium prevents the backtracking of the substrate polypeptide and enhances export by conferring directionality towards the outside of the bacterium, a process known as the ratchet mechanism [34]. Those findings indicate that there are complex intermediate protein-protein interactions involved in secretion. The fundamental source of these complications is the C-terminal localization of the secretion signal; a complete translation is required prior to secretion. Since the export passage is too narrow for a folded globular protein to pass, there must be some mechanism that retains the synthesized substrate polypeptide relatively unfolded and translocation-competent [35].

There is a paradox, in that GFP-LARD3 had a native fluorescence while being most efficiently secreted. The preserved fluorescence implies a stably-folded GFP, while the high secretion efficiency indicates the intrinsically-disordered, relatively unfolded, translocation-competent state of the fusion protein [36]. The more compactly the GFP was folded, the harder it become for the fusion protein to pass through the MFP-OMP channel, decreasing the secretion efficiency. This seeming contradiction implied a more complex state of the substrate polypeptide during secretion, such as being locally folded at the fused domain (GFP in this case) and unfolded at the secretion signals. Also, upon binding extracellular Ca^+2^ ions, the secreted GFP-LARD fusion proteins would probably assume folded C-terminal regions, but the folding of the GFP in the extracellular medium is uncertain because only low fluorescence was detected in the extracellular medium. In the case of EGF, EGF-LARD3 shows normal EGF function after secretion [37]. The structure and function of secreted proteins were not sure but the proteins were assumed to be folded after the secretion but possibly failed to fold into normal structure.

## Conclusions

The RTX motif directly linked with fusion protein enhanced secretion and preserved the fluorescence of GFP. The 4 RTX repeats in TliA were not strictly required for secretion as LARD4, which is deficient of 2 out of 4 RTX repeats, was still secreted. However, as LARD3 showed, the secretion was most efficient in the presence of all 4 RTX repeats without any additional N-terminal residues. LARD3 was a minimal region in TliA capable of exporting GFP and maintaining its fluorescence. LARD3 was recently used to make a transgenic probiotic microbe secreting EGF for enhanced would healing [37]. LARD3 presents a suitable tool to produce useful recombinant proteins extracellularly in *E. coli* or in *P. fluorescens*, and even more, to design an *in situ* protein manufacturing factory utilizing living microbes in producing and secreting proteins of interest.

## Methods

### Bacterial strains and plasmids

*E. coli* XL1-Blue, *E. coli* MM294, and *P. fluorescens* SIK W1 (KCTC 7689) were used as hosts for DNA manipulation and gene expression. *E. coli* MM294 (F^–^*endA1 hsdR17* (r_K_^–^m_K_^+^) *glnV44 thi-1 relA1 rfbD1 spoT1)* was used as expression host because GFP was expressed well to show strong fluorescence. This strain is very close to wild type *E. coli* K-12 [31]. *P. fluorescens* SIK W1 *ΔtliA*, a knock-out mutant lacking the genomic TliA, was constructed in the authors’ laboratory. Plasmids pKK223-3, pDSK-519, and pACYC-184 were used as expression vectors. The plasmids were introduced into *E. coli* cells by heat transformation and into *P. fluorescens* cells by electroporation or conjugation using *E. coli* F-positive strain S17-1 as a donor.

### Expression of fusion proteins

*E. coli* was grown in Luria-Bertani (LB) at 37°C for one day in an orbital shaker at 150 rpm. For the expression of fusion proteins, *E. coli* was grown in 2 ml medium in a 15 ml culture tube. Isopropyl β-d-1-thiogalactopyranoside (IPTG) was added at 0.05 mM for the expression of pKK223-3-derived plasmids which is under *tac* promoter. For dual plasmid expression, 50 μg/ml ampicillin was used to maintain the plasmids derived from pKK223-3, and 34 μg/ml chloramphenicol was used to maintain the pACYC-184 derivatives. *P. fluorescens* was grown in LB or 2× LB media at 25°C for 2 days. The 2× LB medium is a doubly concentrated LB medium used for specific growth of *P. fluorescens*. *P. fluorescens* was grown in 8 ml medium in a 20 ml glass tube at 150 rpm to reduce the degradation of proteins by aeration [38]. pDSK519-derived plasmids were under *lac* promoter but IPTG was not used because expression of genes was constitutive in *P. fluorescens*[38]. For one vector system in *P. fluorescens*, 30 μg/ml kanamycin was used to maintain the pDSK-519 derivatives.

### Plasmid construction

The plasmids used in this research are summarized in Table 1. The GFP gene from pGFPuv (Clontech, Mountain View, CA) and LARD coding sequences from pTOTAL were PCR amplified using primers with *Eco*RI/*Xba*I and *Xba*I/*Hind*III sites, respectively. Factor Xa protease cleavage site (IEGR) was added between the GFP and LARDs as a linker by attaching the corresponding oligonucleotide to the primers. The GFP and LARD sequences were inserted respectively into *Eco*RI-*Xba*I and *Xba*I-*Hind*III sites of pKK223-3 downstream of the *tac* promoter to construct pGFP-LARD1 to 5. The EGF gene was PCR-amplified using EGF-containing plasmid pGEM-hEGF and EGF-fusion proteins were constructed in a similar manner as GFP-fusion proteins. The *prtDEF* gene from *Erwinia chrysanthemi* was inserted into *Sac*I-*Nde*I site of pACYC-184 to construct pEcPrtDEF. Those plasmids were introduced simultaneously into *E. coli* XL1-Blue or MM294 via heat transformation. The *tliA* and *tliDEF* genes were inserted into pDSK-519 under the *lac* promoter to construct pDSK-TliA and pDSK-TliDEF (abbreviated as pDX throughout this paper), respectively. In addition, the coding sequences for GFP, TliA, GFP-LARD3, and GFP-TliA were inserted into *Kpn*I-*Sac*I site of pDX downstream of the *tliDEF* to construct pDX-GFP-223, pDX-TliA, pDX-GFP-LARD3, and pDX-GFP-TliA, respectively. Those plasmids were introduced into *E. coli* S17-1 cells by heat transformation prior to conjugation.

**Table 1:** The plasmids used in this research

**Plasmids**	**Characteristics**	**Reference**
pKK223-3	Cloning vector, Ap^r^	Amersham
pACYC-184	Cloning vector, Cm^r^	New England Biolabs
pDSK-519	Cloning vector, Km^r^	[39]
pGFPuv	*gfp*	Clontech
pTOTAL	*P. fluorescens tliA* operon	[10]
pGFP-TliA	*gfp-tliA* in pKK223-3	This study
pGFP-LARD1 to 5	*gfp-lard1* to *5* in pKK223-3	This study
pEGF-LARD1 to 5	*egf-lard1* to *5* in pKK223-3	
pEcPrtDEF	*prtDEF* in pACYC-184	This study
pDSK-TliA	*tliA* in pDSK-519	This study
pDSK-TliDEF (pDX)	*tliDEF* in pDSK-519	This study
pDX-GFP-223	*gfp* and *tliDEF* in pDSK-519	This study
pDX-TliA	*tliA* and *tliDEF* in pDSK-519	This study
pDX-GFP-TliA	*gfp-tliA* and *tliDEF* in pDSK-519	This study
pDX-GFP-LARD3	*gfp-lard3* and *tliDEF* in pDSK-519	This study

### LARD design

TliA has a lipase activity domain in residues 1–268, a hinge region in residues 269–278, a secretion/chaperone domain in residues 279–476, and a calcium-binding domain in residues 373–417 [11]. The location of PCR amplification sites for the design of LARDs was determined according to the three-dimensional structure of TliA predicted by the SWISS-MODEL structural modeling program at http://swissmodel.expasy.org/[30]. Appropriate PCR primers were constructed to acquire five different LARDs each elongating from residues 303, 337, 373, 407, and 442 to the C-terminus. The oligonucleotides for the Factor Xa protease cleavage site were added to the primers, and the primers were used to fuse the LARDs to the C-terminus of GFP by means of ligation to construct GFP-LARD fusion proteins.

### SDS-PAGE and western blotting

Cells were grown in the growth conditions described in the *Bacterial strains, plasmids, and growth conditions* section until they reached the stationary phase, which took approximately 24 hours for *E. coli* and 48 hours for *P. fluorescens*. The cultures were separated into pellets and supernatants by double centrifugation from which the expression and secretion of the GFP-LARD fusion proteins were analyzed separately. Gel electrophoresis was undertaken according to Laemmli [40]. Fifteen micro-liters of cell extract or supernatant, equivalent to 15 μl OD_600_ ~ 2.5 culture broth (0.0375 OD_600_ equivalent), was loaded on 10% (v/v) SDS-PAGE, and western blotting was performed as described previously [11], using anti-LARD or anti-GFP primary antibodies. The purified TliA (99 μg/ml) was diluted to 3.3 or 1.4 μg/ml and used as the internal standard for western blotting.

### Estimation of GFP fluorescence

*E. coli* cells harboring pGFP-223, pGFP-LARD1 to 5, or pGFP-TliA was grown in 3 ml LB (50 μg/ml ampicillin) at 37°C for 18 hr or at 25°C for 36 hr. Different amounts of each culture were centrifuged, washed, and resuspended to meet OD_600_ = 2. One hundred microliters of the re-suspended culture was added to Greiner 96 well Fluotrac^TM^ for fluorescence measurement. Fluorescence was estimated with excitation at 485 nm and emission at 535 using a Tecan Genios Pro multifunction microplate reader. The relative fluorescence of each culture was obtained by comparing to that of the culture harboring pGFP-223. *E. coli* cells expressing different GFP fusion proteins were streaked on an LB plate, grown for 1 or 2 days, and photographed under long-wavelength UV light.

### Construction of the *P. fluorescens* knock-out mutant, *ΔtliA*

*P. fluorescens* SIK W1 *ΔtliA* was constructed using *sacB*, a levansucrase-encoding gene from *Bacillus subtilis*, as a selection marker. Levansucrase catalyzes levan synthesis through transfructorylation of sucrose [41] and confers lethality to a variety of gram-negative bacteria [42]. The *tliA* gene was PCR amplified from pTOTAL and inserted into *BamH*I*/Hind*III site of pK18*mobsacB*, a vector with *sacBKm*^*R*^*,* and a *mob* factor [43]. The plasmid was digested by *Xho*I and *Sal*I, which were in the middle of *tliA*, and ligated to make an internal 512 bp-deficient mutant of *tliAΔtliA*. A constructed vector pK*tliAXS* with *ΔtliA* was transferred from *E. coli* S17-1 to *P. fluorescens* SIK W1 by conjugation using the *mob* factor. Since the replication origin of pK18*mobsacB* is inactive in *P. fluorescens*[43], the recombinant *P. fluorescens* was cultured with kanamycin and sought for the resistant strains with pK*tliAXS* inserted into the genome. That mutant strain conveyed sucrose sensitivity along with kanamycin resistance, implying a single crossover event between the intrinsic *tliA* and *ΔtliA*. In order to induce another crossover that replaced *tliA* with *ΔtliA* and eliminated *sacB* and *Km*^*R*^ from the genome, a single colony of the mutant strain was grown in a nonselective LB medium and spread onto a 10% sucrose LB plate. *P. fluorescens ΔtliA* was selected as the colonies that grew only on the nonselective LB plate and had no lipase activity on a tributyrate plate.

## Abbreviations

TliA, Thermostable lipase A; ABC, ATP binding cassette; T1SS, Type 1 secretion system; LARD, Lipase ABC transporter recognition domain; GFP, Green fluorescent protein; EGF, Epidermal growth factor; TliDEF, ABC transporter for TliA; PrtDEF, ABC transporter in Erwinia chrysanthemi; RTX, Repeat in toxins.

## Competing interests

The authors declare that they have no competing interests.

## Authors’ contribution

YP played leading role in writing the manuscript. YM played major role in design of experiments and western blot results. JR contributed to primer design, vector constructions and most experiments. NK and HC participated all experiments. JHA designed experiments and interpreted results. All authors read and approved the final manuscript.
